# Total Hemi-overgrowth in Pigmentary Mosaicism of the (Hypomelanosis of) Ito Type: Eight Case Reports: Erratum

**DOI:** 10.1097/01.md.0000482689.36580.ef

**Published:** 2016-04-18

In the article “Total Hemi-overgrowth in Pigmentary Mosaicism of the (Hypomelanosis of) Ito Type Eight Case Reports”,^1^ which appeared in Volume 95, Issue 10 of *Medicine*, Dr. Ruggieri's name was originally misspelled. The article has since been corrected online.
